# Is Serum VEGF-A Level an Indicator of Early-Onset Poststroke Depression?

**DOI:** 10.3390/medicina60111828

**Published:** 2024-11-07

**Authors:** Emine Yildirim Uslu, Sevler Yildiz

**Affiliations:** 1 Department of Physical Medicine and Rehabilitation, Elazığ Fethi Sekin City Hospital, Elazığ 23280, Turkey; e.yildirim9346@gmail.com; 2 Department of Psychiatry, Elazığ Fethi Sekin City Hospital, Elazığ 23280, Turkey

**Keywords:** poststroke depression, vascular endothelial growth factor, early-onset poststroke depression, lesion location

## Abstract

*Background and Objectives*: Poststroke depression (PSD) is a psychiatric complication occurring after a stroke, and is known to negatively impact quality of life. In the present study, the possible relationship between serum vascular endothelial growth factor (VEGF-A) levels and early-onset PSD, as well as the predictive value of serum VEGF-A levels for early-onset PSD, were investigated. *Materials and Methods*: The study included 88 individuals diagnosed with acute ischemic stroke (AIS). Demographic data, clinical characteristics, and serum VEGF-A levels were recorded, and radiological images were examined to determine the lesion locations. The National Institutes of Health Stroke Scale (NIHSS), Montreal Cognitive Assessment (MoCA), and Hamilton depression scale (HAMD-17) were administered to the patients. Furthermore, serum VEGF-A levels were measured in all participants. *Results*: Although the body mass index (BMI) and VEGF-A levels were similar between the groups, MoCA scores were lower [(19.2 ± 4.4) vs. (22.3 ± 3), *p* = 0.001] and NIHSS scores were higher [18 (8–28) vs. 14 (3–24), *p* = 0.006] in individuals with PSD than in those without it. When the patients with PSD were categorized into three groups, patients with severe PSD had higher NIHSS scores [26 (23–27) vs. 15 (8–23), *p* = 0.006] and lower MoCA scores [(14.3 ± 1) vs. (20.9 ± 3.8), *p* = 0.005] than those with mild PSD. Moreover, VEGF-A levels and lesion localization were similar between mild, moderate, and severe PSD groups (*p* = 0.130). The MoCA score was negatively (r = −0.498, *p* < 0.001) correlated and the NIHSS score was positively correlated (r = 0.497, *p* < 0.001) with the HAMD-17 score. *Conclusions*: Our findings suggest that longitudinal studies in large cohorts including healthy control groups are needed to examine the possibility of using serum VEGF-A level as a marker for predicting early-onset PSD.

## 1. Introduction

Poststroke depression (PSD) is one of the most common complications following a stroke, affecting approximately 20–60% of stroke patients [1,2]. PSD is characterized by depressive mood, apathy, changes in sleep patterns, feelings of worthlessness, and anhedonia. It is known to be one of the poor prognostic factors of stroke, negatively affecting functionality and quality of life [3]. Additionally, it is associated with an increased risk of stroke recurrence and mortality [4,5]. Therefore, identifying PSD, managing it effectively, and intervening in a timely manner is crucial. PSD can occur at different stages following a stroke. PSD occurring within two weeks after the onset of acute stroke is defined as early-onset PSD [6,7]. Furthermore, it is known that depressive symptoms are more aggressive in early-onset PSD compared to late-onset PSD [8].

Multiple social, psychological, and biological factors are thought to play a role in the development of PSD. Sociodemographic factors such as age, female gender, low educational level, neurotic personality, unemployment, and lack of social support have been found to be associated with PSD [1,9,10,11]. Moreover, it has been suggested that the level of functional impairment and severity of paralysis are also closely associated with PSD [1,12]. The relationship between lesion location and PSD has also been a topic of research. In addition to studies associating left hemisphere lesions and frontal lobe or basal ganglia involvement with PSD [13,14,15], there are also studies suggesting that lesion localization is not associated with PSD [16,17,18]. Some researchers have proposed that cognitive impairment resulting from stroke may increase the risk of PSD [19]. However, it is also believed that cognitive impairment may stem from PSD [20]. The overlap between cognitive impairment and depressive symptoms can lead clinicians to overlook the diagnosis of PSD, particularly in the early stages [21]. The relationship between PSD and poststroke cognitive impairment has not been adequately clarified.

The biological mechanisms proposed for PSD are extremely diverse. These mechanisms include decreased monoamine levels, glutamate-mediated excitotoxicity, abnormal neurotrophic response, and inflammatory factors [1]. There is also increasing evidence regarding the involvement of neuroimmune inflammatory activation in the mechanisms of depression and PSD. It has been reported that abnormal inflammatory responses alter neurotransmission and lead to significant dysfunction in the brain [22,23,24]. Additionally, genetic polymorphisms related to inflammation and the ischemic cascade are thought to be involved in the etiology of PSD [25,26]. Neurogenesis has also been a topic of interest for researchers studying PSD. It has been shown that brain-derived neurotrophic factor (BDNF) plays an important role in the maintenance of neurogenesis and plasticity, and low BDNF levels are associated with PSD [27]. Along with BDNF, other molecules known to be involved in neurogenesis include insulin-like growth factor-1 (IGF-1), fibroblast growth factor 2 (FGF-2), and vascular endothelial growth factor (VEGF-A) [28].

VEGF-A is a vascular growth factor and permeability regulator, also known as vascular permeability factor. Circulating levels of it increase in response to inflammation and hypoxic processes. It also binds to vascular endothelial cells and promotes their growth, playing a role in hypoxia-induced neovascularization [29]. It has two important receptors, those being VEGFR-1 and VEGFR-2. It is known that angiogenic response and vascular permeability are induced by binding to the VEGFR-2 receptor under hypoxic conditions [30]. In addition to its proangiogenic activity, VEGF-A has neurotrophic and neuroprotective potential in the peripheral and central nervous systems [31]. VEGF-A produced by ependymal cells has been shown to stimulate neuronal precursor proliferation in the central nervous system [32]. It plays an important role in the proliferation and survival of neural stem cells and in the migration and maturation of neural progenitor cells [33]. The dysregulation of neurotrophic factors like VEGF-A is thought to play a significant role in the pathophysiology of depression. Matsuno et al. [34] demonstrated in their studies on mice that VEGF plays an important role in the pathogenesis of depression by increasing blood–brain barrier permeability, suggesting that VEGF-A receptor inhibition could be a potential treatment strategy. Viikki et al. [35] demonstrated a significant relationship between VEGF gene polymorphisms and treatment-resistant depression. Włodarczyk et al. [36] proposed that low VEGF-A levels are associated with the improvement of depressive symptoms.

Early-onset PSD cases may differ from late-onset PSD in terms of mechanisms and symptomatology. Although the mechanisms have not been elucidated, it is known that depressive symptoms are more severe and long-term outcomes are worse in early-onset PSD compared to late-onset PSD [8]. Therefore, identifying predictors of early-onset PSD is of great value. Various molecules have been investigated for this purpose. Geng et al. [37] found that low glutamate levels after a stroke were associated with early-onset PSD. Deng et al. [38] suggested that high levels of homocysteine and fibrinogen could be independent risk factors for early-onset PSD, and could be used as predictive indicators. However, there is insufficient research in the literature regarding whether serum VEGF-A levels after a stroke serve as a predictor for the development of early-onset PSD. This study aimed to investigate the relationship between VEGF-A levels in stroke patients and depression scores in the early stages.

## 2. Materials and Methods

### 2.1. Participants and Procedure

This case-control study was conducted at the Fethi Sekin City Hospital, Elaziğ, Turkey. The study protocol was approved by the Research Ethics Committee of the Firat University of Medicine, Elaziğ, Turkey (date: 20 May 2024/no: 24287). The study was conducted in accordance with the 2013 Declaration of Helsinki, and written informed consent was obtained from all participants. G*Power 3.1.9.2 statistical power analysis was used to estimate the sample size. The calculation ensured 95% statistical power at a significance level (alpha error probability) of 0.05. To achieve sufficient statistical power, a total of 88 samples were included, with a projected required sample size of 76.

Inclusion criteria were as follows: over 18 years of age and diagnosed with acute ischemic stroke by computed tomography (CT) or magnetic resonance imaging (MRI) within 24 h of hospitalization. Exclusion criteria were as follows: patients with impaired consciousness and dysarthria/aphasia who were unable to answer the questions on the scales used in the study; patients with a history of cognitive impairment, such as dementia; patients with a history of other neurological diseases and psychiatric diseases; a history of malignancy; liver and kidney failure; and patients with an intellectual disability in the prestroke period, according to the medical history and anamnesis taken.

### 2.2. Clinical Variables, Neuroimaging, and Blood Biomarker Analysis

Because of the importance of starting rehabilitation early [39], patients were referred to us in the acute rehabilitation phase, after hemodynamic and clinical stabilization was achieved within the first 72 h after stroke. Demographic characteristics, medical history, and current clinical status of patients who experienced ischemic stroke were assessed. Blood pressure was measured for all participants, and routine blood tests were conducted. Blood pressure (systolic/diastolic) was measured within the first 4 h after admission. MRI scans, including T1-weighted, T2-weighted, and diffusion-weighted imaging, performed within 24 h after admission, were reviewed to determine the location of the brain infarct. Infarct localization was recorded as anterior circulation (middle cerebral artery, anterior cerebral artery) or posterior circulation (vertebrobasilar artery, posterior cerebral artery).

Blood samples from all participants were collected within 72 h poststroke, between 08:00 and 08:30, after fasting for at least 8 h. For routine hemogram tests, 3 mL of EDTA-anticoagulated whole blood was used [automated hematology analyzer, Beckman Coulter DXH 800, Brea, CA, USA]. A routine biochemical analysis was performed using an automated analyzer (Beckman AU 5800, Brea, CA, USA). For VEGF-A analysis, 3 mL of blood was collected into a biochemistry tube. The blood collection tube was allowed to stand vertically for an average of 30–40 min. Subsequently, the tube was centrifuged at 3000 rpm for 10 min. After centrifugation, the serum remaining at the top was transferred to Eppendorf (Hamburg, Germany) tubes with a pipette. The serum samples were stored at −80 °C until the day of the study. Samples transferred to the laboratory under suitable conditions were analyzed using the ELISA method.

The ELISA kit uses the competitive-ELISA principle. The micro ELISA plate provided in this kit has been precoated with VEGF-A. During the reaction, VEGF-A in the sample or standard competes with a fixed amount of VEGF-A on the solid-phase supporter for sites on the biotinylated detection Ab that is specific to VEGF-A. Excess conjugate and unbound sample or standard are washed from the plate, and avidin that were conjugated to horseradish peroxidase (HRP) were added to each microplate well and incubated. Then, a TMB substrate solution was added to each well. The enzyme–substrate reaction was terminated by the addition of stop solution, and the color change is measured spectrophotometrically at a wavelength of 450 nm ± 2 nm. The concentration of VEGF-A in the samples was then determined by comparing the OD of the samples to the standard curve.

Functional status, cognition, and depression were assessed on the 14th day after stroke in patients who had not received any psychiatric medication or therapy support after stroke. Functional status was assessed by a Physical Medicine and Rehabilitation specialist, using the National Institutes of Health Stroke Scale (NIHSS). Patients were evaluated by a psychiatrist according to the Diagnostic and Statistical Manual of Mental Disorders, 5th edition (DSM-5) [40]. Cognitive status was assessed with the Montreal Cognitive Assessment test, and the presence/severity of depression was assessed with the Hamilton depression rating scale (HAMD-17). Patients were divided into mild, moderate, and severe early-onset PSD groups according to the HAMD-17 score.

### 2.3. Scales Used in the Study

Hamilton Depression Rating Scale (HAMD-17): HAMD-17 is a 17-item test used by physicians to measure the severity of depression in patients. It queries the depressive symptoms experienced in the last week. Based on the HAMD-17 score, a score of 7–17 indicates mild depression, 18–23 indicates moderate depression, and 24 and above indicates severe depression. The scale has been validated and is known to be reliable in the Turkish population [41,42].

National Institutes of Health Stroke Scale (NIHSS): This scale consists of items that provide a quantitative measurement of the basic components of a standard neurological examination. It assesses the neurological functions of stroke patients and offers insights into long-term prognosis. The total NIHSS scores can range from 0–42. According to the NIHSS, a score of 0 indicates normal function, 1–4 indicates mild stroke, 5–14 indicates moderate stroke, 15–20 indicates moderate–severe stroke, and ≥21 indicates severe stroke [43].

Montreal Cognitive Assessment (MoCA): Developed as a rapid screening test for mild cognitive impairment, the MoCA evaluates attention and concentration, executive functions, memory, language, visual construction skills, abstract thinking, calculation, and orientation. The score range on the MoCA is 0–30; a score of 21 points or higher is considered normal [44,45].

### 2.4. Statistical Analysis

The Statistical Package for the Social Sciences (SPSS) version 21.0 was used for the statistical analyses. Baseline characteristics of the sample were described as follows: frequency and percentage for categorical variables, mean and standard deviation (SD) for normal continuous variables, and median, minimum, and maximum for non-normal continuous variables. Normality was assessed using the Kolmogorov–Smirnov test and histograms. First, patients were divided into 2 groups as either PSD or non-PSD. The statistical analysis was conducted using appropriate tests based on the data distribution to analyze PSD vs. non-PSD, and mild PSD vs. moderate PSD vs. severe PSD. The normally distributed numerical parameters were compared using a student’s *t*-test or one-way analysis of variance (ANOVA) in groups, while those with non-normal distributions were analyzed using a Mann–Whitney U-test or a Kruskal–Wallis test. The categorical variables were compared by using Chi-squares or Fisher’s Exact tests where appropriate. The strength of the relationship between the numerical data of the participants was carried out using Spearman’s or Pearson’s correlation coefficient. As both parameters were normally distributed, the correlation coefficients and their significance were calculated using the Pearson test, while investigating the associations between non-normally distributed and/or ordinal variables, and the correlation coefficients and their significance were calculated using the Spearmen test. A %5 type-I error level was used to infer statistical significance. Statistical difference was considered when *p*-value < 0.05.

## 3. Results

A total of 88 participants were included in the study, with a mean age of 61.50 ± 4.7 years. Sixty-seven (76.1%) patients had pre-existing hypertension (HT), twenty-six (29.5%) had diabetes mellitus (DM), and twenty-one (23.9%) had coronary artery disease. As summarized in Table 1, participants with PSD and non-PSD were comparable for age, gender, incidence of hypertension, type 2 DM, and a history of coronary heart disease. Although body mass index (BMI) and VEGF-A levels were similar between the groups, MoCA scores were lower [(19.2 ± 4.4) vs. (22.3 ± 3), *p* = 0.001] and NIHSS scores were higher [18 (8–28) vs. 14 (3–24), *p* = 0.006] in individuals with PSD than in those without it. There was no difference between the groups regarding complete blood count parameters and cholesterol levels. The baseline demographic and laboratory characteristics of the patients are summarized in Table 1 and Table 2.

When the patients with PSD were categorized into three groups, patients with severe PSD had higher NIHSS scores [26 (23–27) vs. 15 (8–23), *p* = 0.006] and lower MoCA scores [(14.3 ± 1) vs. (20.9 ± 3.8), *p* = 0.005] than those with mild PSD. VEGF-A levels and lesion localization were similar between mild, moderate, and severe PSD groups (Table 3).

The relationship between the participants’ numerical data is depicted in Table 4. The MoCA score was negatively correlated and the NIHSS score was positively correlated with the HAMD-17 score. There was no correlation between VEGF-A levels and HAMD-17 scores, age, or BMI.

## 4. Discussion

The present study examined whether serum VEGF-A levels predict early-onset PSD in patients after ischemic stroke, as well as the relationship between serum VEGF-A levels and clinical variables of stroke patients. It was observed that as the severity of the disease increased, the rate of disability increased, and cognitive capacity decreased in patients with early-onset PSD. No correlation was observed among the severity of the disease, depression, and serum VEGF-A levels in patients with early-onset PSD.

In the present study, early-onset PSD was diagnosed in 38.6% of patients 2 weeks after the onset of the stroke. A meta-analysis conducted in 2023 reported that poststroke depression typically occurs within 3 months after the stroke, with a high risk of persistence; the prevalence was noted to be 24% (95% CI 21–28), based on clinical interviews, and 29% (95% CI 25–32), based on rating scales [46]. In the present study, it was observed that patients in the severe early-onset PSD group demonstrated higher NIHSS scores and lower MoCA scores compared to those with mild and moderate PSD. Furthermore, a correlation was observed between the severity of depression and the NIHSS and MoCA scores. This suggests that the severity of early-onset PSD may vary depending on the disabilities and cognitive changes caused by the stroke. Although there are studies reporting that women are more likely than men to develop depression after a stroke [47], there is also research suggesting there are no gender differences among patients regarding the development of PSD [48]. A 2020 study on stroke patients found that age did not create a difference in the risk of early-onset PSD [49]. Similarly, no gender differences were observed among the patients in the present study, indicating that the level of physical limitation poststroke equally affects functionality in similar age groups, indicating that gender does not create a difference in the development of depression. There is evidence of a prognostic effect of smoking on stroke severity and disability [50]; however, no differences were observed in the development of early-onset PSD among stroke patients in the present study. Although hypertension, DM, and coronary artery disease are risk factors for ischemic stroke [51,52], no differences were observed in the development of early-onset PSD. Chen et al. noted in their study that there were no differences between patients diagnosed with PSD, and those who were not, in regard to the presence of diabetes, hypertension, hyperlipidemia, alcohol, and smoking. Although it is known that these systemic diseases associated with atherosclerosis are related to depression, further studies are needed to explain the causal relationship between early-onset PSD and these conditions [53]. Currently, there is no definitive relationship established between the location of lesions and PSD [54], but some studies have reported that the severity of early-onset PSD is associated with the location of the lesion [55]. Moreover, differences in the presentation of psychiatric disorders poststroke may be influenced by the dominant hemisphere of the brain [56]. Supporting the literature, the present study showed no relationship between lesion location and the development of early-onset PSD. These inconsistent results may stem from the effects of hemispheric roles on mental states. We believe that further studies using functional imaging methods will be useful to evaluate the brain region causing stroke and the related lesion with parameters, such as reperfusion level and duration.

VEGF-A plays a key role in the neuronal and vascular effects in stroke due to its trophic and protective effects on neurons, as well as its role in atherosclerosis and angiogenesis [57,58]. Zhang et al. conducted a study on 225 stroke patients and found that serum VEGF-A levels were significantly elevated in stroke patients (40.01 ± 16.48 pg/mL) compared to controls (32.98 ± 10.35 pg/mL). These results suggested that elevated serum VEGF-A is an independent predictor of adverse outcomes in minor ischemic strokes [59]. In another study, it was reported that there were different VEGF-A levels and receptors in various stages of strokes, showing that there are increased levels of VEGF-AR-2 in the hyperacute and acute phases of ischemic stroke, whereas elevated VEGF-A and decreased VEGF-AR-1 levels were identified in the early subacute phase. In this study, it was interpreted that tissue changes and circulatory disorders in the hyperacute and acute phases of stroke indicate ongoing regeneration, including neoangiogenesis, in the early subacute periods. In this study, VEGF-A levels were measured as 669 pg/mL in the early subacute phase after stroke, whereas, in our study, it was 649.95 pg/mL in the PSD group and 582.77 pg/mL in the non-PSD group [60]. High VEGF-A levels are frequently observed in conjunction with ischemic attacks, leading to studies that consider serum VEGF-A levels as a potential biomarker for stroke. However, recent studies suggest that serum VEGF-A levels may not be significantly associated with the diagnosis of ischemic stroke [61]. A study conducted in 2023 aimed to examine potential prognostic markers for recovery in poststroke rehabilitation, investigating VEGF-A gene expression and serum VEGF-A levels. It reported statistically significant changes in clinical parameter predictions during the rehabilitation process, along with VEGF-A protein and mRNA expression. Consequently, it was suggested that serum VEGF-A levels could be used to indicate the level of cognitive improvement in poststroke patients [36]. Similarly, a study conducted in Indonesia on stroke patients in a similar age group to those in the present study found that high serum VEGF-A levels were associated with larger infarct volumes and cognitive impairment after stroke [62].

Early recognition of PSD, which is known to negatively affect the mortality and quality of life of stroke patients, is important. In recent years, research on identifying biomarkers for early-onset PSD has gained impetus. Deng et al. [38] suggested that elevated homocysteine and fibrinogen levels may be used as predictive indicators for early-onset PSD. However, there is insufficient research in the literature regarding whether serum VEGF-A levels after stroke serve as a predictor for the development of early-onset PSD. Duman and Monteggia [63] proposed that stress leads to a decrease in neurotrophins like VEGF-A, suggesting that the reduction in neurotrophin levels may cause atrophy in certain limbic structures, which could result in depressive symptoms, under the neurotrophic depression model. Although there are conflicting results regarding VEGF-A levels in patients with depression, VEGF-A is believed to be important in the etiopathogenesis of depression [64]. In a meta-analysis examining the effect of VEGF-A, a neurotrophic factor, on major depressive disorder (MDD), it was reported that the patient group had high VEGF-A levels, although there were conflicting results [65]. Fornaro et al. reported that there was no difference between the VEGF-A levels of MDD patients and controls [66]. Carvalho et al. observed lower serum VEGF-A levels in MDD patients compared to healthy controls [67]. In a study evaluating cognitive functions in patients with Alzheimer’s disease, it was shown that serum VEGF-A levels increased as the severity of the disease increased, and this increase represented the endogenous response generated by Alzheimer’s disease [68]. In Alzheimer’s patients with depression, serum VEGF-A levels were found to be higher compared to those without depression, emphasizing the relationship between disease pathogenesis and elevated VEGF-A, an angiogenic cytokine [69]. Kotan et al. reported no difference in serum VEGF-A levels between patients with major depressive disorder and a control group, but noted that VEGF-A levels decreased as the severity of depression increased [70]. The current literature includes various results concerning the relationship between serum VEGF-A levels, depression, and disease severity. In the present study, serum VEGF-A levels of patients diagnosed with PSD in the second week after ischemic stroke were compared to those without the diagnosis. No significant differences were found between the two groups. When classifying the severity of depression in early-onset PSD patients according to the HAMD-17, no significant difference was observed between depression severity and serum VEGF-A levels. Although most of the studies in patients with depressive disorders suggest that patients have high VEGF-A levels [65], there are also studies that do not support this [66,67]. This study, in which we examined the relationship between serum VEGF-A levels and depression severity in early-onset post-ischemic stroke patients, is the first study to examine this relationship, and different results may be due to many factors, such as a different study design, patient population, age and gender distribution, comorbidities, and the sample source (plasma, serum).

Previous studies have shown that VEGF-A levels increase with age [71]. It has also been reported that serum VEGF-A levels in patients with depression are not influenced by BMI [72]. In the present study, it was observed that serum VEGF-A levels did not change based on the patients’ BMI and age. Thus, we believe that it may be appropriate to re-evaluate this relationship in another study including a healthy control group, taking into account the increase in comorbid conditions with age.

The limitations of the study include the lack of a control group consisting of healthy subjects with similar sociodemographic and clinical characteristics to the patient group, the cross-sectional nature of the study, the relatively small sample size, and the insufficient number of patients in the distribution according to disease severity. The fact that the diagnosis of early-onset PSD was made by a psychiatrist according to a structured clinical interview and the HAMD-17 scale, but not evaluated according to neurotransmitter levels, is also among the limitations. In addition, since the samples were taken in the first 72 h after stroke, although the patients did not receive psychiatric medication and therapy support during this period, the fact that they received stroke treatment (antiaggregant, antihypertensive, etc.) may have affected serum VEGF levels.

## 5. Conclusions

In conclusion, our study suggests that longitudinal studies in larger sample sizes are needed to determine whether peripheral serum VEGF-A levels are appropriate markers for predicting early-onset PSD and determining the severity of depression in these patients. To the best of our knowledge, this is the first study evaluating VEGF-A levels in patients with early-onset PSD. If the possible relationship between the serum VEGF-A level and early-onset PSD discussed above is supported by additional evidence, it is important in terms of early diagnosis and risk stratification of the disease to ensure that early interventions can be made. We believe that the findings of our study will shed light on future studies.

## Figures and Tables

**Table 1:** Clinical characteristics of the participants.

	Non-PSD (*n* = 54)	PSD (*n* = 34)	*p*
Gender			0.362
Female, n (%)	26 (48.1)	13 (38.2)	
Male, n (%)	28 (51.9)	21 (61.8)	
Age ± SD (years)	60.9 ± 4.4	62.4 ± 5	0.136
Smoking, n (%)			0.763
No	30 (55.6)	20 (58.8)	
Yes	24 (44.4)	14 (41.2)	
Hypertension, n (%)			0.953
No	13 (24.1)	8 (23.5)	
Yes	41 (75.9)	26 (76.5)	
Diabetes Mellitus, n (%)			0.616
No	37 (68.5)	25 (73.5)	
Yes	17 (31.5)	9 (26.5)	
Coronary Artery Disease, n (%)			0.649
No	42 (77.8)	25 (73.5)	
Yes	12 (22.2)	9 (26.5)	
Antiplatelet medication, n (%)			0.649
No	42 (77.8)	25 (73.5)	
Yes	12 (22.2)	9 (26.5)	
Antihypertensive medication, n (%)			0.953
No	13 (24.1)	8 (23.5)	
Yes	41 (75.9)	26 (76.5)	
Antistatin medication, n (%)			0.811
No	44 (81.5)	27 (79.4)	
Yes	10 (18.5)	7 (20.6)	
Antidiabetic medication, n (%)			0.616
No	37 (68.5)	25 (73.5)	
Yes	17 (31.5)	9 (26.5)	
Localization of the lesion, n (%)			0.648
Posterior/PCA	16 (29.6)	6 (17.6)	
Posterior/VBA	6 (11.1)	5 (14.7)	
Anterior/ACA	4 (7.4)	3 (8.8)	
Anterior/MCA	28 (51.9)	20 (58.8)	
Hemispheric dominance, n (%)			0.119
Non-dominant	21 (38.9)	19 (55.9)	
Dominant	33 (61.1)	15 (44.1)	
BMI ± SD (kg/m^2^)	26.9 ± 2.3	27.6 ± 2.7	0.193
VEGF-A, (pg/mL) median (min–max)	649.95 (17.43–2014.96)	582.77 (23.83–2072.07)	0.684
MoCA ± SD	22.3 ± 3	19.2 ± 4.4	0.001 *
NIHSS, median (min–max)	14 (3–24)	18 (8–28)	0.006 *

(PSD: poststroke depression; PCA: posterior cerebral artery; VBA: vertebrobasilar artery; ACA: anterior cerebral artery; MCA: middle cerebral artery; BMI: body mass index; VEGF: vascular endothelial growth factor; MoCA: Montreal Cognitive Assessment; NIHSS: National Institutes of Health Stroke Scale); * statistically, differences were found between the groups.

**Table 2:** Laboratory parameters of the participants.

	Non-PSD (*n* = 54)	PSD (*n* = 34)	*p*
WBC, median (min–max)	7.38 (2.60–18.28)	7.15 (4.30–13.20)	0.901
Neutrophil, median (min–max)	4.01 (1.90–10.04)	3.71 (2.12–12.78)	0.262
Lymphocyte, median (min–max)	2.36 (0.48–5.27)	2.58 (0.50–7.40)	0.461
Triglyceride, (mg/dL) median (min–max)	146 (50–381)	130 (62–276)	0.253
HDL ± SD (mg/dL)	51 ± 12.7	53.2 ± 10.8	0.391
LDL ± SD (mg/dL)	124.3 ± 26.9	138.4 ± 37.2	0.060

(PSD: poststroke depression; WBC: white blood cell; HDL: high-density lipoprotein; LDL: low-density lipoprotein).

**Table 3:** Characteristics of the participants with PSD.

	Mild PSD (*n* = 21)	Moderete PSD (*n* = 9)	Severe PSD (*n* = 4)	*p*
VEGF-A, (pg/mL) median (min–max)	874.92 (23.83–2072.07)	136.33 (35.97–1399.35)	446.36 (81.03–2007.40)	0.130
NIHSS, median (min–max)	15 (8–23) *	21 (12–28)	26 (23–27) *	0.006
MoCA ± SD	20.9 ± 3.8 *	17.3 ± 4.4	14.3 ± 1 *	0.005
Localization of the lesion				0.195
Posterior/PCA	5 (23.8)	0 (0)	1 (25)	
Posterior/VBA	1 (4.8)	3 (33.3)	1 (25)	
Anterior/ACA	1 (4.8)	1 (11.1)	1 (25)	
Anterior/MCA	14 (66.7)	5 (55.6)	1 (25)	

* statistically, differences were found between the groups; (PSD: poststroke depression; VEGF: vascular endothelial growth factor; NIHSS: National Institutes of Health Stroke Scale; MoCA: Montreal Cognitive Assessment; PCA: posterior cerebral artery; ACA: anterior cerebral artery; MCA: middle cerebral artery).

**Table 4:** Relationship between the numerical data of the participants.

		HAMD-17	VEGF
MoCA	r	−0.498	0.075
	*p*-value	<0.001	0.485
NIHSS	r	0.497	−0.031
	*p*-value	<0.001	0.777
VEGF-A (pg/mL)	r	−0.088	
	*p*-value	0.412	
Age (years)	r	0.127	−0.170
	*p*-value	0.238	0.113
BMI (kg/m^2^)	r	0.119	−0.058
	*p*-value	0.271	0.589

(HAMD-17: Hamilton Depression Rating Scale; VEGF: vascular endothelial growth factor; MoCA: Montreal Cognitive Assessment; NIHSS: National Institutes of Health Stroke Scale; BMI: body mass index).

## Data Availability

The datasets used and analyzed in the current study are available from the corresponding author upon reasonable request.
